# Totally endoscopic surgical resection for a blood cyst originated from mitral valve: a case report

**DOI:** 10.1186/s13019-021-01535-6

**Published:** 2021-06-07

**Authors:** Xin Zhang, Lin Zhang, Lianggang Li, Tong Ren, Shengli Jiang

**Affiliations:** Department of Cardiovascular Surgery, Chinese PLA General Hospital, No.28. Fuxing Road, Haidian District, Beijing, 100853 China

**Keywords:** Thoracoscopic surgery, Minimally invasive surgery, Cardiac tumors, Valvular tumors, Case report

## Abstract

**Background:**

Intracardiac blood cysts are very rare primary cardiac tumors. Blood cysts originated from the mitral valve represent a minority of cases, and previous cases have been mainly treated with conventional surgery through median sternotomy. When the tumor involves heart valves and histopathological diagnosis remains unknown preoperatively, minimally invasive surgical resection of an intracardiac tumor can be challenging, especially through an endoscopic approach. We herein present the first case of successful surgical resection for a rare mitral valve originated blood cyst in a minimally invasive, totally thoracoscopic approach.

**Case presentation:**

An apparently healthy 38-year-old male presented to his local hospital with six months history of palpitation and exertional dyspnea. Transthoracic echocardiography showed a mobile round cystic mass inside the left ventricle, attached to the anterolateral papillary muscle and chordae tendineae of the mitral valve. The local doctor diagnosed an intracardiac tumor and suggested a surgical resection through median sternotomy. However, the patient refused to have a sternotomy. He was then referred to us seeking minimally invasive surgery. We assessed the location, appearance and relationship to nearby structures of the tumor with echocardiography, and made a diagnosis of a suspected primary cystic intracardiac tumor. Since we had enough experience of totally endoscopic mitral surgery, our surgical plan was to resect the tumor in the aid of thoracoscopy, and manage the possible deformation and dysfunction of the cardiac structure if necessary. Using femoro-femoral cannulation and cardiopulmonary bypass, we successfully resected the tumor through a thoracoscopic approach in a closed chest, and well preserved the subvalvular structure and valvular function. Postoperative recovery was quick and uneventful. Pathologic diagnosis confirmed a simple blood cyst.

**Conclusions:**

Surgical resection is warranted for symptomatic cases of intracardiac blood cysts. With prudent preoperative diagnosis and comprehensive surgical plan, we believe the thoracoscopic approach is a safe, curative and viable alternative for complete resection of cardiac valvular tumors.

**Supplementary Information:**

The online version contains supplementary material available at 10.1186/s13019-021-01535-6.

## Background

Intracardiac blood cysts are uncommon congenital pseudoneoplasms. Although small blood cysts can occasionally be found in postmortem examinations in infants, large cysts in adult patients are extremely rare, and very few cases have been reported to date [1, 2]. Due to its sparsity, there is no consensus addressing the management of intracardiac blood cysts. Surgery for previous cases of intracardiac blood cysts had been mainly performed through conventional sternotomy. A blood cyst originated from the mitral valve has to our knowledge never been surgically resected with a totally endoscopic, closed-chest approach before. Here we present the first case treated with totally thoracoscopic surgery.

## Case presentation

A 38-year-old male complained of palpitation and exertional dyspnea on his first visit to a local hospital in February. He never had a fever, arthralgias, weight loss or fatigue, nor did he have a personal or relevant family history of cardiovascular disease or neoplasms. A grade II/6 pansystolic heart murmur was heard during physical examination. Transthoracic echocardiography (TTE) revealed a large mass in the left ventricle, attached to the anterolateral papillary muscle and chordae tendineae. The local doctor made a preliminary diagnosis of suspected intracardiac fibroelastoma or myxoma, and suggested surgical resection for this intracardiac neoplasm through conventional median sternotomy. However, the patient was deterred by the invasiveness of sternotomy, and refused to have surgery in his local hospital. In May, as he learned about our experience in minimally invasive cardiac surgery, he came to our center.

**Fig. 1:**
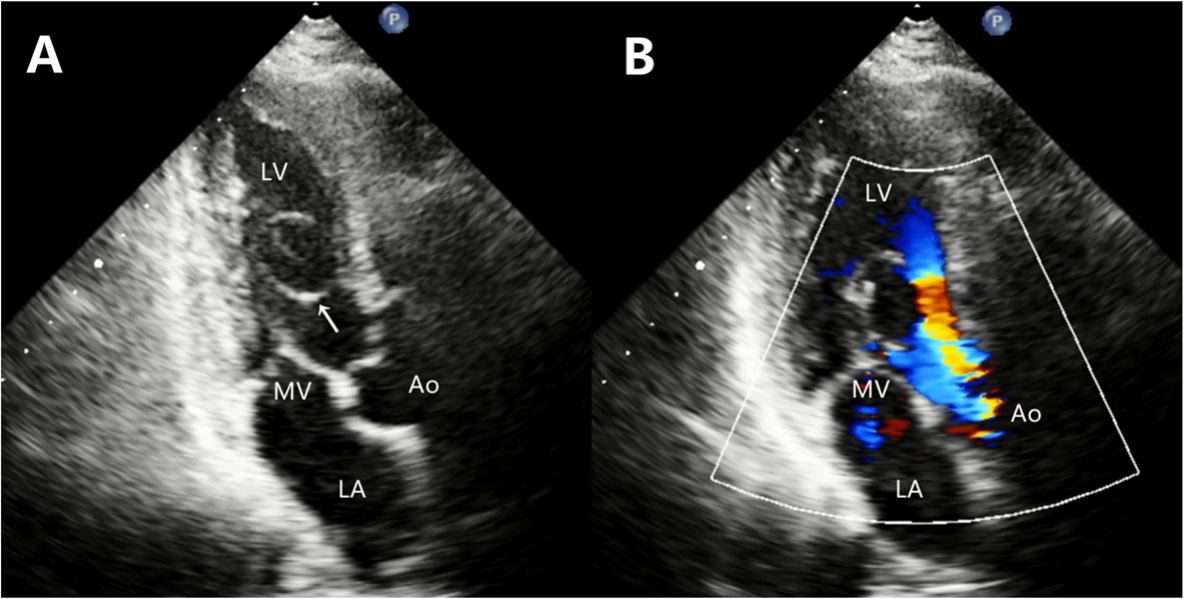
The preoperative echocardiography demonstrated (**A**) a highly mobile, hyperechogenic lesion (arrow) attached to the anterior mitral leaflet of the mitral valve, (**B**) the accelerated blood flow of the left ventricular outflow tract with mitral regurgitation. LV, left ventricle; Ao, aorta; MV, mitral valve; LA, left atrium

We reexamined the patient. The TTE confirmed a mobile round cystic mass inside the left ventricle. The mass had a hyperechogenic wall and hypoechogenic content and was attached to the anterior mitral leaflet (Fig. 1A). In systole, the mass prolapsed into the left ventricular outflow tract (LVOT) with accelerated peak velocity (2.5 m/s), which led to mitral regurgitation (Fig. 1B). The LVOT appeared normal without subvalvular ridge or asymmetric septal hypertrophy. Complementary thoracic and abdominal computed tomographic (CT) scans did not show any evidence of the existence or potential spread of noncardiac neoplasms. All laboratory examinations including tumor markers were unremarkable.

Non-invasively distinguishing aggressive from non-aggressive intracardiac tumors is an important clinical challenge. Preoperative biopsy for histopathological diagnosis is inapplicable to intracardiac tumors. In this case, fortunately, the TTE provided significant information on morphology, localization, biological characteristics, as well as tumor vascularity-perfusion. The mass had a smooth surface, clear border, and low echodensity. No imaging evidence showed any features of malignancy like vascularity-perfusion, invasion or filtration into the cardiac structure, permeation to the great vessels, or pericardial effusion. So, there was a fairly high probability that this mass would be benign. We made a diagnosis of a primary intracardiac cystic tumor according to the obtained information, and decided to resect the tumor.

Generally, a benign intracardiac tumor in this location and size can be accessed via trans-aortic or mitral orifice approach through mini-thoracotomy. Since we have enough experience of totally endoscopic mitral surgery, we decided to perform the operation in a totally thoracoscopic approach, and manage the possible deformation and dysfunction of the cardiac structure if necessary.

**Fig. 2:**
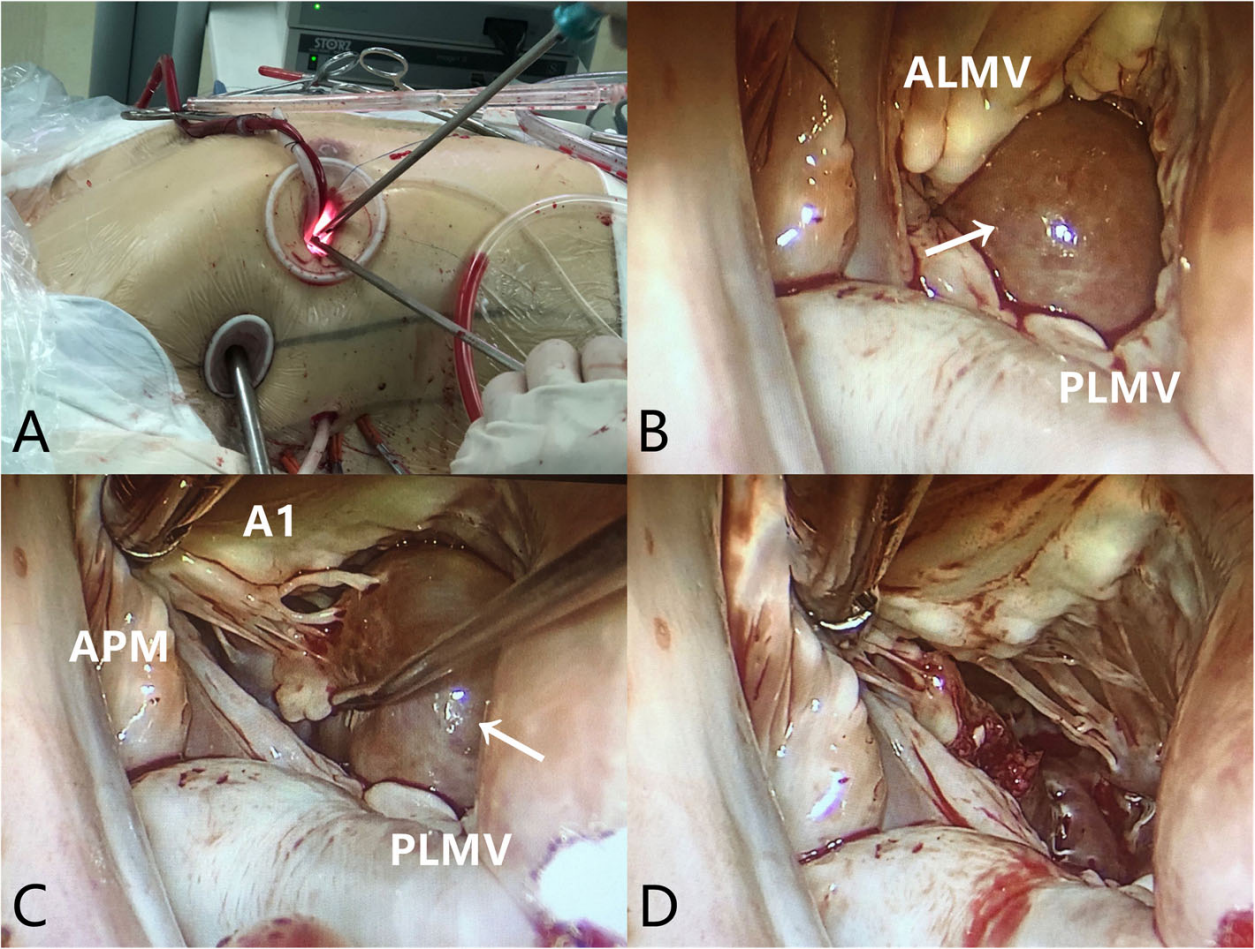
Intraoperative images **A** The surgical approach. **B** Exposure of the mitral orifice and the mass (arrow). **C** A mass (arrow) was partly attached to the chordate structure of the anterior papillary muscle and A1 area via two pedicles. **D** Exploration of mitral leaflets and subvalvular apparatus. ALMV, anterior leaflet of the mitral valve; PLMV, posterior leaflet of the mitral valve; APM, Anterior papillary muscle; A1, A1 area of ALMV


Tumor resection was planned and performed through a totally endoscopic, video-assisted approach with thoracoscopy and femoral cannulation (Supplemental Video). The cardiopulmonary bypass and the surgical approach were established as the conventional endoscopic mitral surgery (Fig. 2A). A 35-mm working port was made in the fourth interspace, lateral to the midclavicular line. After deflation of the right lung, two additional ports were then placed: a camera port through the anterior axillary line in the fourth interspace, and an 8-mm port in the lateral fifth interspace for a transthoracic Chitwood clamp (Scanlan International, Minneapolis, Minn). We used the Chitwood clamp to occlude the ascending aorta and administrated the antegrade cardioplegia. A left atriotomy was performed, and the atrial retractor was applied. The mitral orifice and the mass were then clearly visualized (Fig. 2B). The mass was oval and reddish, partly attached to the chordate structure of anterior papillary muscle and A1 area of anterior mitral leaflet via two pedicles (Fig. 2C). We completely excised the mass through the roots of the pedicles, and then carefully cauterized the margin of excision. The size and structure of the mitral leaflets and subvalvular apparatus appeared to be normal. No surgical injuries were found during further exploration (Fig. 2D). The saline test showed no mitral regurgitation.

**Additional file 1: Video S1**. Totally thoracoscopic resection of a blood cyst originated from the mitral valve.

**Fig. 3:**
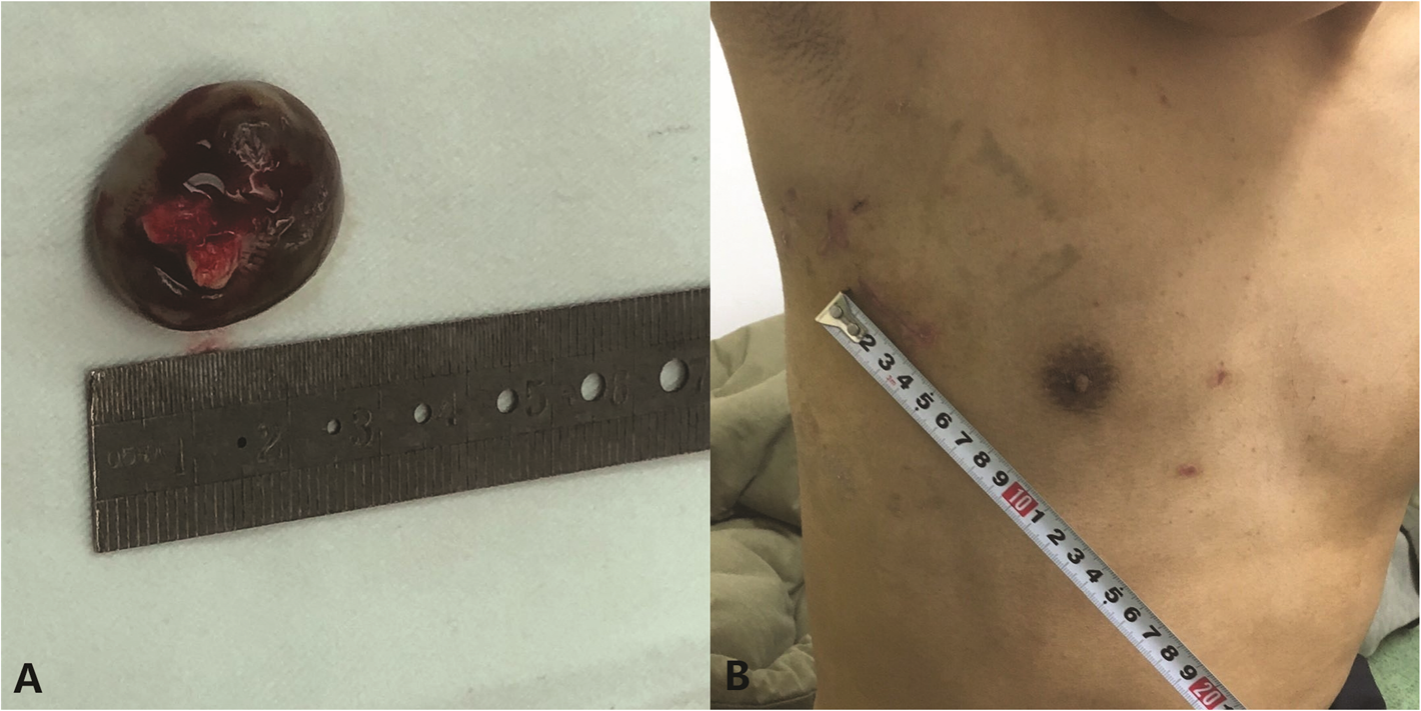
**A** The excised mass (25 mm*20 mm*20 mm). **B** Postoperative scars at a two-week follow-up

The excised mass was 25 mm*20 mm*20 mm in size (Fig. 3A). Pathologic diagnosis confirmed a simple blood cyst, consistent with blood-filled space lined with a single layer of endothelium. The patients had an uneventful recovery. He was able to walk on the first postoperative day and was discharged three days after the operation. The patient was symptom-free and working full time during follow-up.

## Discussion and conclusions

The intracardiac blood cyst was first reported by Elsässer in 1844. Blood cysts are typically lined by flattened endothelial cells and filled with nonorganized blood. These lesions can occasionally be found during autopsy in fetuses or infants, arise characteristically from the mitral and tricuspid valves [3]. Although acquired blood cyst has been reported [4, 5], the majority of cases were of congenital origin. Inflammation, anoxia, hemorrhagic diathesis, vagal stimulation, invaginations of the endothelium, and alterations during valve development are suspected causes [6]. In adults, however, blood cysts are rare and often found incidentally. Researchers did not build any consensus on clinical management. Scattered studies have suggested conservative management for asymptomatic cases [7]. Since the hemodynamic implication of intracardiac blood cysts may cause dyspnea, chest pain, syncope, stroke, or even coronary artery embolism, surgical resection is warranted for symptomatic cases.

Minimally invasive cardiac surgery (MICS) is being applied with increasing frequency in all areas of cardiovascular surgery, and cardiac tumors are no exception. However, experience is generally confined to benign tumors. This is perhaps due to both the difficulty of preoperative pathologic diagnosis and the different adoption of MICS between cardiac centers. Since invasive heart biopsy procedure is usually inapplicable for intracardiac tumors, surgical management for cardiac tumors mainly relies on preoperative differential diagnosis with cardiovascular imaging. Although TTE has offered sufficient basis for us to diagnose and make a surgical plan in this individual case; for common cases, TEE, contrast-enhanced cardiac CT, cardiac magnetic resonance, or even 18F-fluorodeoxyglucose positron emission tomography should be considered with discretion in preoperative evaluation, to facilitate in planning the most appropriate therapeutic strategy for cardiac tumors. To our knowledge, this is the first reported case that had an intracardiac blood cyst surgically resected with a totally endoscopic, closed-chest approach. Previous cases involving mitral valve or causing LVOT obstruction were treated with conventional surgery through a sternotomy [1, 2, 8]. As we have gained experience of totally thoracoscopic mitral valve surgery through hundreds of cases before this one [9], we become more skilled in manipulating the subvalvular apparatus of the mitral valve through a totally endoscopic approach and rarely convert to median sternotomy or thoracotomy. So we chose left atrial-ventricular valve access to complete this procedure. For intracardiac tumors of similar size and location, trans-aortic access should be considered as a viable alternative approach. If a tumor causes LVOT obstruction, then mini-thoracotomy should be considered, especially when the tumor has a larger size. The incision, access, and cardioplegic techniques depend mainly upon the size, location, and involvement of the tumor. Surgeons need to be both skilled and flexible in decision-making. Experience from this single medical center suggests, with prudent preoperative diagnosis and comprehensive surgical plan, the valvular tumor could be safely and completely resected in a minimally invasive approach.

This case additionally benefited from the totally thoracoscopic approach in the following aspects. Firstly, the endoscopic approach provides direct and targeted access to the mitral orifice and the tumor, allowing excellent visualization of the surgical target. The magnification of thoracoscopy enhances scrutiny into the subvalvular apparatus and facilitates a precise dissection at the appropriate layer. Meticulous removal of the mass can be achieved without fragmentation. Besides, since preoperative images well delineated the tumor anatomy and valve involvement, our policy is to perform a full-layers resection of the tumor whenever possible and manage the valvular derangement and dysfunction. This approach even allows a resection and reattachment of the anterior portion of the mitral valve through the access of mitral orifice for exposure, if indicated intraoperatively. Further mitral valve repair could also be performed easily as usual. Moreover, the thoracoscopic approach features a small skin incision (Fig. 3B), sternum preservation, less drainage, and transfusion. These attribute to a quicker recovery, yielding both cosmetic and functional advantages.

In conclusion, a rare blood cyst originated from the mitral valve was successfully resected through a totally thoracoscopic approach. Surgical resection is warranted for symptomatic cases of intracardiac blood cysts. This case exemplifies the expanding potential of thoracoscopic cardiac surgery. If performed adequately, we believe the thoracoscopic approach is a viable and safe alternative for the complete removal of cardiac valvular tumors.

## Data Availability

All data generated or analyzed are included in this article.

## Abbreviations

TTE: Transthoracic echocardiography
LVOT: Left ventricular outflow tract
CT: Computed tomographic
MICS: Minimally invasive cardiac surgery
